# JNK kinase regulates phosphorylation of HCoV-229E nucleocapsid protein

**DOI:** 10.1038/s44298-025-00152-7

**Published:** 2025-09-18

**Authors:** Yannick Brüggemann, Toni Luise Meister, Natalie Heinen, Emely Richter, Saskia Westhoven, Michael Poppe, Mohammed Samer Shaban, Leyla Sirkinti, Maximilian Nocke, Daniel Todt, Stephanie Pfaender, Michael Kracht, Eike Steinmann

**Affiliations:** 1 Department of Molecular and Medical Virology, Ruhr University Bochum, Bochum, Germany; 2 Institute for Infection Research and Vaccine Development (IIRVD), Centre for Internal Medicine, University Medical Centre Hamburg-Eppendorf (UKE), Hamburg, Germany; 3 Department for Clinical Immunology of Infectious Diseases, Bernhard Nocht Institute for Tropical Medicine, Hamburg, Germany; 4 German Centre for Infection Research (DZIF), Partner Site Hamburg-Lübeck-Borstel-Riems, Hamburg, Germany; 5 Research Unit Emerging Viruses, Leibniz Institute of Virology (LIV), Hamburg, Germany; 6 Rudolf Buchheim Institute of Pharmacology, Justus Liebig University, Giessen, Germany; 7 Department of Translational and Computational Infection Research, Ruhr University Bochum, Bochum, Germany; 8 European Virus Bioinformatics Center (EVBC), Jena, Germany; 9 University of Lübeck, Institute of Virology and Cell Biology, Lübeck, Germany; 10 German Centre for Infection Research (DZIF), External Partner Site, Bochum, Germany

**Keywords:** SARS-CoV-2, Viral host response, Virus-host interactions

## Abstract

Identifying common host factors essential for the replication cycles of human coronaviruses (HCoV) could help uncover potential therapeutic targets. Mitogen-activated protein kinases (MAPKs) regulate critical cellular signaling pathways. Among them, c-Jun N-terminal kinases (JNK) are activated in response to diverse environmental stresses, including viral infections. However, the relevance of the JNK pathway for host responses and replication of HCoV infections has remained elusive. Using live-cell microscopy, quantitative immunofluorescence and immunoblotting, we found that JNK is specifically activated in cells infected with HCoV-229E and plays a crucial role in mediating the phosphorylation of the viral nucleocapsid (N) protein, an essential step required during the viral replication cycle. Consequently, pharmacological inhibition of JNK kinase activity impeded HCoV-229E as well as SARS-CoV-2 infection. Given the conservation of phosphorylation sites within the nucleocapsid protein across coronaviruses, inhibitors targeting these N protein kinases, such as JNK, may hold therapeutic promise as broad-spectrum CoV antivirals.

## Introduction

The emergence and reemergence of coronaviruses as significant human pathogens pose persistent threats to global public health, as evidenced by the devastating impact of outbreaks such as severe acute respiratory syndrome coronavirus (SARS-CoV), Middle East respiratory syndrome coronavirus (MERS-CoV)^1^, and severe acute respiratory syndrome coronavirus 2 (SARS-CoV-2), which causes coronavirus disease 2019 (COVID-19)^2^. Additionally, the four common human coronaviruses (229E, HKU1, NL63, and OC43) are responsible for 15% to 30% of common cold cases in adults and can lead to severe illness in high-risk individuals, such as infants, the elderly, and immunocompromised patients^3^. Understanding the intricate interplay between coronaviruses and their host cells is essential for elucidating viral pathogenesis and developing novel therapeutic strategies^4–6^. Central to this endeavor is the characterization of host signaling pathways exploited by coronaviruses to facilitate their replication and evade host immune responses^6^. The c-Jun N-terminal kinases (JNK) pathway regulates multiple cellular processes such as proliferation, apoptosis, and immune responses^7^. JNK belongs to the family of mitogen-activated protein kinases (MAPKs), which are key players in evolutionarily conserved signaling cascades that transduce extracellular stimuli into diverse cellular responses^8–10^. Upon activation by various stressors, including pro-inflammatory cytokines, environmental toxins, and viral infections, JNK kinases phosphorylate downstream substrates, including transcription factors such as c-Jun, leading to the modulation of gene expression and cellular function^11^. Accumulating evidence has linked the JNK pathway to the pathogenesis of numerous viral infections, highlighting its potential as a target for antiviral intervention^12^. In particular, JNK activation has been shown to favor replication of HIV^13^, herpes simplex virus^14^, rotavirus^15^, dengue virus^16^, influenza A virus^17^ and SARS-CoV^18^. However, despite the growing body of research implicating the involvement of JNK kinases in viral infections, comprehensive studies delineating the precise mechanisms by which coronaviruses exploit the JNK pathway, particularly HCoV-229E, remain limited. In this study, the activation and requirement of JNK during the replication cycle of HCoV-229E were examined.

## Results

### HCoV-229E infection promotes JNK phosphorylation and signaling

**Fig. 1:**
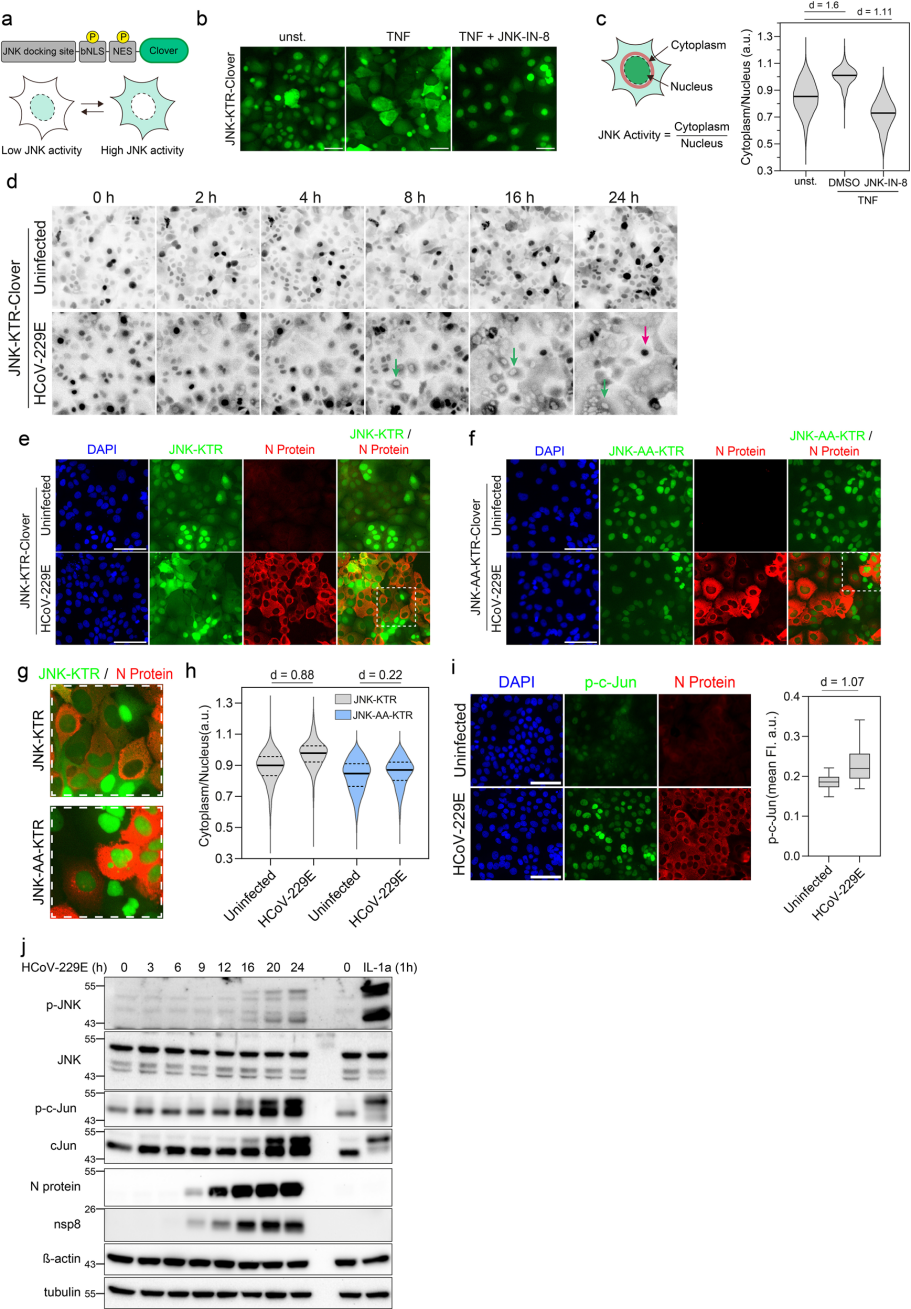
HCoV-229E infection promotes JNK phosphorylation and signaling. **a** Schematic of the phosphorylation-mediated translocation of the JNK kinase translocation reporter (KTR). **b** Huh7 cells stably expressing JNK KTR-Clover were stimulated for 2 h with 100 ng/mL TNF in control cells treated with DMSO or cells pretreated with 10 µM JNK-IN-8. **c** Left: Method used to obtain the cytoplasmic to nuclear ratio of JNK KTR (see “Methods”). Right: JNK activity in Huh7 cells stably expressing JNK KTR-Clover after 2 h stimulation with 100 ng/mL TNF in control cells treated with DMSO or cells pretreated with 10 µM JNK-IN-8 (625 to 1028 cells per condition). **d** Time-lapse of Huh7 cells stably expressing JNK KTR-Clover in uninfected control cells (top) and upon infection with HCoV-229E (bottom) at the indicated times. JNK-KTR translocation in infected cells (green arrows) and non-infected bystander cells (purple arrow). Huh7 cells stably expressing JNK KTR-Clover (**e**) or JNK-AA-KTR-Clover (**f**) upon infection with HCoV-229E and uninfected control cells were stained for the HCoV-229E nucleocapsid. Nuclei were stained with DAPI. **g** Insets of selected cells from (**e**) and (**f**). **h** KTR translocation for JNK KTR-Clover (blue) or JNK-AA-KTR-Clover (gray) in uninfected control cells and upon HCoV-229E infection. Median displayed as solid line and quartiles displayed as dashed lines (2401 to 6562 cells per condition from three independent experiments). **i** Right: Huh7 cells upon infection with HCoV-229E and uninfected control cells were stained for the HCoV-229E nucleocapsid and phosphorylated c-Jun (p-c-Jun). Nuclei were stained with DAPI. Left: Mean nuclear p-c-Jun fluorescence intensities in single cells (12,034 to 17,819 cells per condition from three independent experiments). **j** Lysates of Huh7 cells after inoculation with HCoV-229E for the indicated time points were immunoblotted for phosphorylated JNK (p-JNK; T183/Y185), JNK, p-c-Jun, c-Jun, HCoV-229E nucleocapsid (N protein), nsp8, ß-actin and tubulin. Lysates of Huh7 cells after 1 h stimulation with 10 ng/mL IL-1α for 1 h were used as control (representative western blot from three independent experiments). Effect sizes in **c**, **h** and **i** were calculated as Cohen’s *d* (*d*).

To probe for JNK kinase activities during HCoV-229E infection in living cells, we adapted an imaging system based on a kinase translocation reporter (KTR)^19^. The JNK KTR consists of a substrate recognition motif (peptides derived from the JNK substrate c-Jun), phosphorylation sites (P sites) located near a nuclear localization sequence (bNLS) and a nuclear export sequence (NES) site and a fluorescent protein (Clover). KTR phosphorylation by JNK suppresses bNLS activity and enhances NES activity, leading to a nucleocytoplasmic shuttling event that can be measured by fluorescence microscopy. After phosphorylation by JNK, the KTR relocates from the nucleus to the cytosol and returns to the nucleus when dephosphorylated (Fig. 1a). We stably transduced Huh7 cells with a JNK-KTR fused to the green fluorescent protein Clover (Fig. 1b). In the absence of external stimuli, the sensor mainly localized towards the nucleus. Stimulation with the prototypical JNK activator tumor necrosis factor-alpha (TNF-α) resulted in a translocation of the sensor from the nucleus towards the cytoplasm due to increased phosphorylation-dependent nuclear export as previously described (Fig. 1b, c). KTR translocation could be prevented by the specific JNK kinase inhibitor JNK-IN-8, demonstrating that the translocation event reflects JNK kinase activity (Fig. 1b, c). JNK kinase activity was quantified based on the ratio of the cytosolic to the nuclear fluorescence intensity values derived from individual cells (cytoplasm/nucleus ratio—Fig. 1c). Overall, these data demonstrate the functionality and specificity of the JNK kinase reporter system in Huh7 cells.

Next, to monitor the activation dynamics of JNK upon infection with HCoV-229E, we performed live cell imaging experiments in Huh7 JNK KTR cells. Following HCoV-229E infection, we observed translocation of the JNK-KTR from the nucleus towards the cytoplasm after approximately 16 h p.i. In particular, cells which underwent syncytia formation showed strong translocation of the reporter construct to the cytoplasm, indicating strong JNK activation. In contrast, the reporter did not translocate towards the cytoplasm in non-infected control cells (Fig. 1d and Supplementary Movie 1). To test if JNK is specifically activated in HCoV-229E infected cells, we performed Immunofluorescence staining of the HCoV-229E N protein in Huh7-JNK-KTR cells 24 h after infection (Fig. 1e). JNK-KTR translocation was restricted to N protein-positive cells, implying JNK activation only in HCoV-229E-infected cells. To exclude potential artifacts in JNK-KTR translocation due to syncytia formation and/or morphological changes of infected cells, we employed a non-phosphorylatable KTR mutant (JNK-KTR-AA). The JNK-KTR-AA construct was strictly localized to the nucleus irrespective of HCoV-229E infection (Fig. 1f). The differential subcellular localization of both constructs upon HCoV-229E infection (Fig. 1g) was further reflected by a strong increase of the cytoplasm/nucleus ratio for the JNK-KTR construct, while the ratio for the JNK-KTR-AA construct remained unchanged (Fig. 1h). Hence, JNK-KTR translocation upon HCoV-229E specifically reflects catalytic activity of endogenous JNK kinase. Accordingly, we further observed phosphorylation of endogenous c-Jun, a member of the downstream JNK transcription factor activator protein-1 (AP-1)^7^, specifically in HCoV-229E-infected single cells (Fig. 1i). These results were corroborated by Western blot analysis, which revealed virus-inducible phosphorylation of several JNK isoforms concomitant with an increase of c-Jun expression and phosphorylation approximately 12–16 h after infection (Fig. 1j). Expression of viral proteins (N protein and nsp8) was already detected 9 h after infection (Fig. 1j). In summary, these results demonstrated progressive activation of the JNK-c-JUN pathway in HCoV-229E-infected cells within 24 h of infection, but not in non-infected bystander cells.

### JNK inhibition prevents HCoV-229E infection

**Fig. 2:**
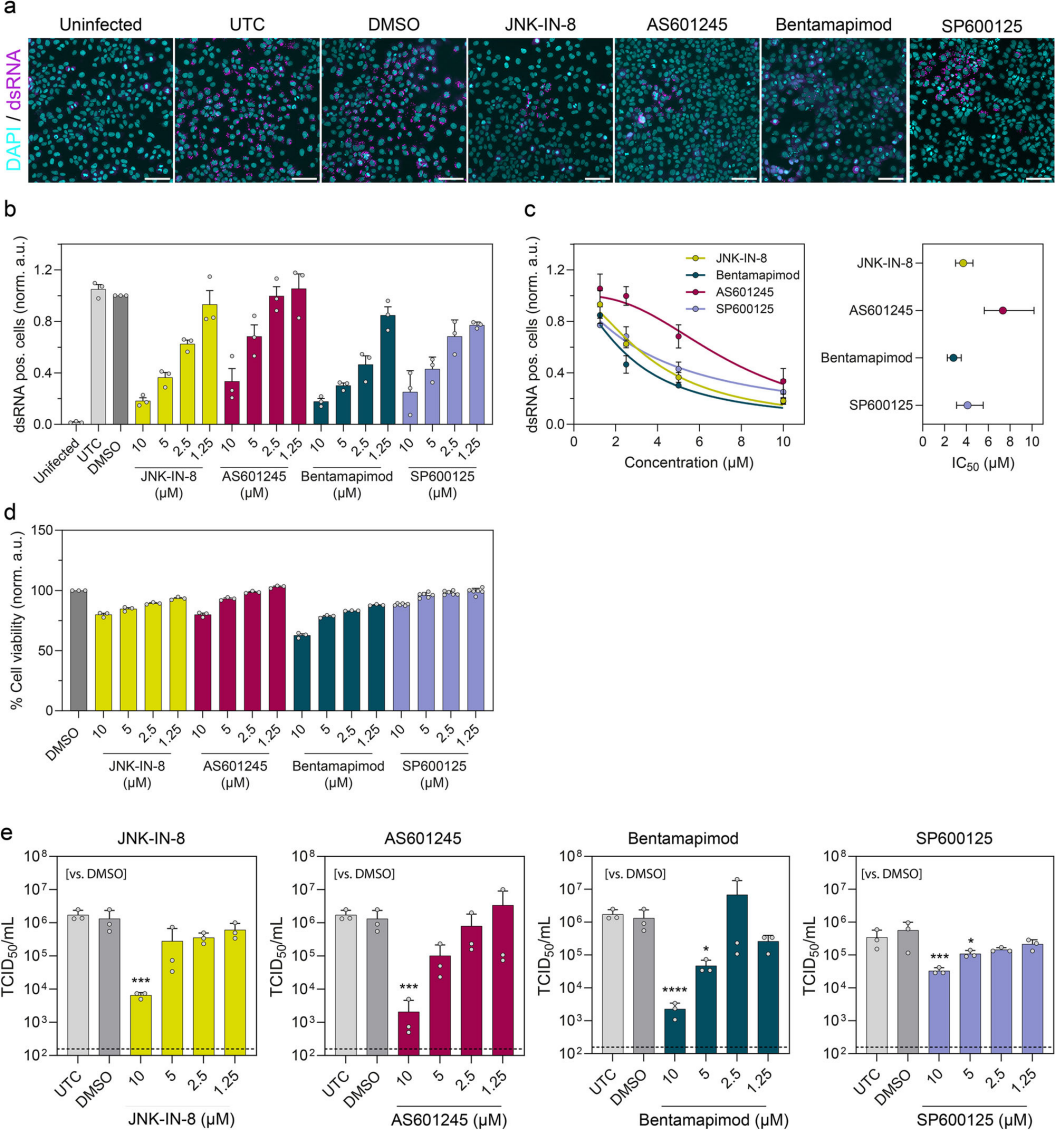
JNK inhibition prevents HCoV-229E infection. **a** Immunofluorescence images of double-stranded RNA (dsRNA) in Huh7 cells 24 h after infection with HCoV-229E (MOI 0.1), either pretreated with different JNK inhibitors (all 10 µM), DMSO, untreated control cells (UTC) and uninfected control cells. Nuclei were stained with DAPI. Scale bars = 100 µm. **b** Quantification of the normalized fraction of dsRNA-positive cells (mean ± SEM, *n* = 3). **c** Left: A non-linear regression model was used to calculate IC_50_ values were calculated using GraphPad Prism. Right: Depicted are the respective IC_50_ values and confidence intervals (CI 95%). **d** Normalized cell viability in percent (%) upon for cells treated with JNK inhibitors, DMSO and untreated control cells (mean ± SEM, *n* = 3). **e** Huh7 cells were either pretreated with different JNK inhibitors in the indicated concentrations, DMSO, or left untreated (UTC) and subsequently inoculated with HCoV-229E (MOI 0.1) for 1 h. Twenty-four hours post-infection, the supernatant was collected and viral titers determined by an endpoint dilution assay and calculated as TCID_50_/mL (mean ± SD, *n* = 3). Dashed lines indicate the lower limit of quantification (LLOQ). Statistical significance was determined using a one-way ANOVA with Dunnett’s post hoc test (****p < 0.0001, ***p < 0.001 and *p < 0.05).

Given the strong activation of JNK upon HCoV-229E infection, we next assessed if inhibition of JNK kinase activity affects HCoV-229E infectivity. To account for potential inhibitor-specific (off-target) effects, we employed four different JNK kinase inhibitors (JNKi), namely JNK-IN-8; AS601245; Bentamapimod and SP600125^20^, which specifically target the ATP-binding pocket of the JNK catalytic domain. Huh7 cells were pretreated with increasing inhibitor dosages for 1 h and subsequently infected with HCoV-229E and stained for double-stranded RNA (dsRNA)—a validated marker of active coronavirus replication^21,22^—to identify infected cells (Fig. 2a). Quantitative immunofluorescence showed a strong dose-dependent decrease in dsRNA-positive cells (Fig. 2a, b). Inhibitor titration revealed only minor changes in IC_50_ values indicating no major differences in the potency among the different JNKi (Fig. 2c). Although all inhibitors reduced cell viability at high concentrations, they impaired cell viability by not more than 20% at concentrations that reduced viral replication by at least 50% (Fig. 2d). In agreement with this, all four inhibitors lowered the production of infectious viruses (TCID50/mL—Fig. 2e) by several orders of magnitude, when used at higher concentrations (>5 µM). Accordingly, siRNA-mediated knockdown of JNK1/2 reduced the production of infectious viruses (Supplementary Fig. 1). Collectively, our data highlight the requirement of JNK activity during HCoV-229E infection.

### The HCoV-229E N protein is phosphorylated in a JNK-dependent manner

**Fig. 3:**
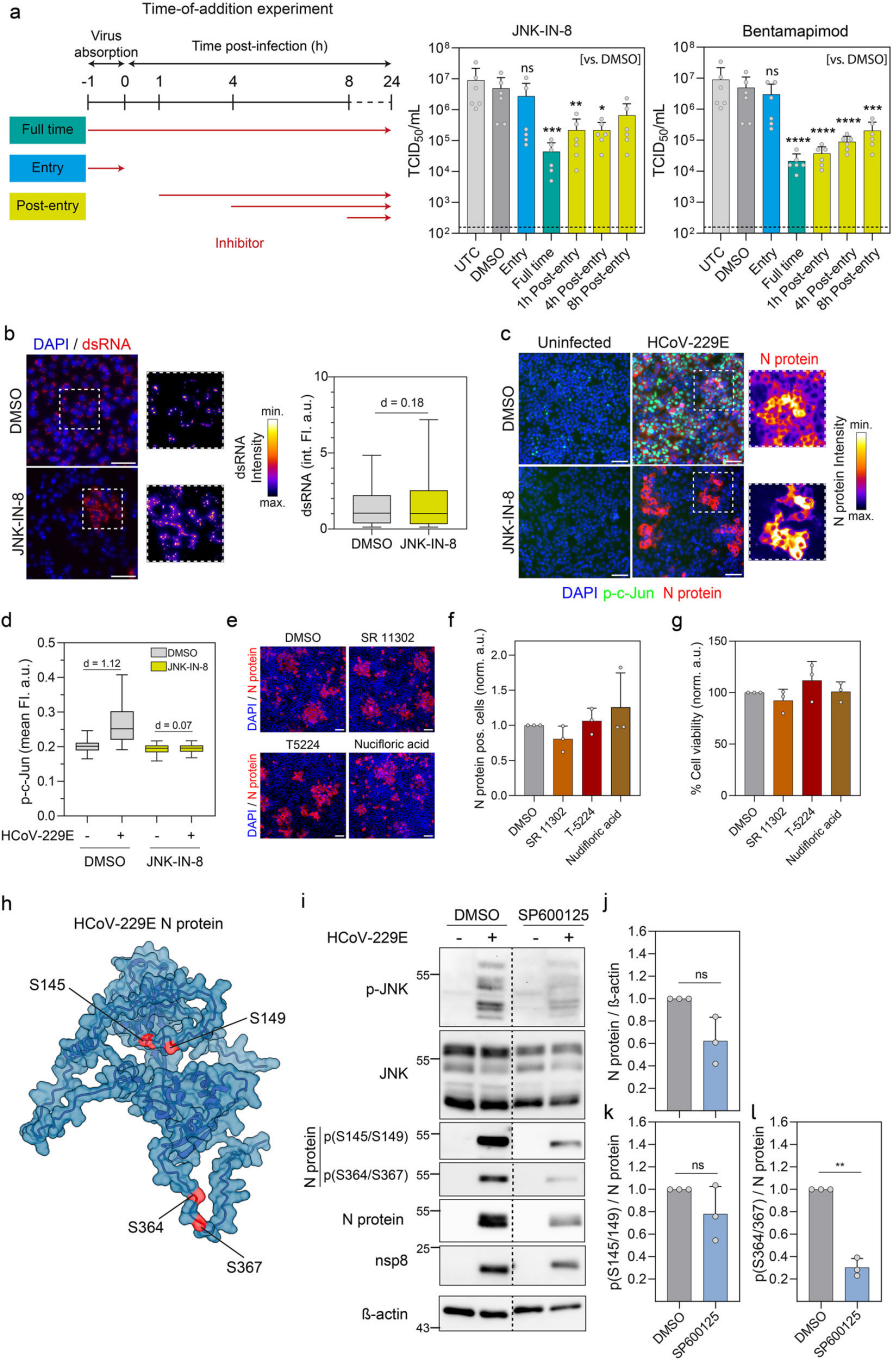
The HCoV-229E N protein is phosphorylated in a JNK-dependent manner. **a** Left: Scheme of the time-of-addition assay. Right: Huh7 cells were inoculated with HCoV-229E (MOI 0.1) for 1 h and treated with either 10 µM of JNK-IN-8 or Bentamapimod, DMSO or left untreated (UTC) and subsequently inoculated with HCoV-229E (MOI 0.1) for 1 h. After 24 h, viral titers were determined by TCID_50_/mL (mean ± SD, *n* = 6). Statistical significance was determined using a one-way ANOVA with Dunnett’s post hoc test (****p < 0.0001, ***p < 0.001, **p < 0.01 and *p < 0.05). **b** Left: Double-stranded RNA (dsRNA) in Huh7 cells 24 h after infection with HCoV-229E (MOI 0.1) upon pretreatment with 10 µM JNK-IN-8 or DMSO. Right: Mean dsRNA fluorescence intensities in single cells (14,196 to 80,374 cells per condition from three independent experiments). **c** Immunofluorescence of HCoV-229E nucleocapsid (N protein) in Huh7 cells in uninfected control cells or 24 h after infection with HCoV-229E (MOI 0.1) upon pretreatment with 10 µM JNK-IN-8 or DMSO. **d** Boxplot of mean nuclear p-c-Jun fluorescence in single cells (1324 to 16,222 cells per condition from three independent experiments). **e** Immunofluorescence of N protein 24 h after infection with HCoV-229E (MOI 0.1) upon pretreatment with AP-1 inhibitors (all 25 µM) or DMSO. **f** Normalized fraction of N protein-positive cells (mean ± SD; *n* = 3). Right: **g** Normalized cell viability in percent (%) upon for cells treated with AP-1 inhibitors (all 25 µM) or DMSO (mean ± SD, *n* = 3). **h** Alpha-fold model of the HCoV-229E nucleocapsid protein. Serine residues (S145/149 and S364/367) are highlighted in red. **i** Lysates of uninfected Huh7 cells upon treatment with DMSO or 20 µM SP600125 and inoculation with HCoV-229E for 24 h were immunoblotted for phosphorylated JNK (p-JNK; T183/Y185), JNK, p-c-Jun, c-Jun, N protein, phosphorylated N protein (S145/S149) and (S364/S367), nsp8 and ß-actin (representative western blot from three independent experiments). Normalized N protein expression (**j**), relative S145/S149 phosphorylation (**k**) and relative S364/367 phosphorylation (**l**) (mean ± SD, *n* = 3). Statistical significance was determined using Welch’s *t*-test (**p < 0.01). All scale bars = 100 µm. Nuclei were stained with DAPI. Effect sizes in **c** and **d** were calculated as Cohen’s *d* (*d*). Dashed lines in **a** and **d** indicate the lower limit of quantification (LLOQ).

To broadly determine which steps of the HCoV-229E replication cycle are affected by JNK, we conducted time-of-addition analysis experiments. We initiated treatment with two different JNKi at various time points during infection (Fig. 3a). (Pre-)application of either JNK-IN-8 or Bentamapimod during the 1-h infection period did not significantly impact the amount of infectious virus produced, suggesting that JNK inhibition does not affect virus attachment or entry into target cells. In contrast, application of either inhibitor after viral entry strongly inhibited virus infection, implying that JNK functions at a post-entry step during the viral replication cycle. We then tested whether JNK activity is required during viral replication by measuring the amount of dsRNA viral RNA in single cells. As previously observed, treatment with JNK-IN-8 greatly reduced the number of infected cells (Fig. 3b). However, quantification of the dsRNA signal in the remaining infected cells upon JNK-IN-8 inhibitor treatment revealed that JNK inhibition had only a weak effect on the amount of dsRNA produced per cell (Fig. 3b). Consistent with this, JNK inhibition did not alter the amount of viral nucleocapsid protein under the same conditions in the remaining infected cells (Fig. 3c). These data suggest that JNK inhibition appears not to modulate viral replication and protein synthesis directly, once a cell is infected. We hypothesized that JNK activates downstream host factors, such as c-Jun, to regulate the (transcriptional) expression of components that are required during other steps of the viral life cycle. As observed previously (Fig. 1i, j), infection with HCoV-229E resulted in a strong increase of c-Jun phosphorylation that was suppressed by JNK-IN-8 treatment (Fig. 3d). However, the inhibition of c-Jun phosphorylation did not affect N protein synthesis in those cells with remaining viral replication, suggesting that the JNK-inhibitory effects described above did not necessarily rely on phosphorylation of canonical c-Jun sites (Fig. 3d). We further tested this notion by means of three different AP-1 inhibitors, including T-5224, which specifically inhibits the DNA binding activity of the c-Fos/c-Jun heterodimer^23^ as well as SR 11302 and 1-Methyl-6-oxo-1,6-dihydropyridine-3-carboxylic acid (Nudifloric acid), which inhibit the AP-1 transcription factor^24,25^. To confirm the activity of the AP-1 inhibitors, we generated an AP-1 reporter construct containing a green fluorescent protein (GFP) under the control of AP-1 response elements and a minimal promoter (Supplementary Fig. 2a)^26^. Treatment with AP-1 inhibitors reduced GFP expression in response to IL-1α stimulation, confirming effective inhibition of AP-1 activity (Supplementary Fig. 2b, c). Importantly, none of the inhibitors altered the number of cells infected with HCoV-229E (Fig. 3e, f), while preserving high levels of cell viability (Fig. 3g). Similarly, stable expression of a dominant-negative Jun variant^27^ (Supplementary Fig. 2d) did not affect HCoV-229E infection (Supplementary Fig. 2e). We therefore concluded that the antiviral effects of JNKi occurred largely independent of the c-Jun/AP-1 pathway.

Ample evidence implies posttranslational modifications in the functionality of viral proteins^28^, with phosphorylation being particularly important^29,30^. While phosphorylation sites have been identified in various viral proteins, the CoV N protein stands out as the most extensively phosphorylated protein^31,32^. To test if JNK mediates the phosphorylation of the viral N protein, we used specific antibodies that were generated to probe for phosphorylated serine 145 and 149 (S145/149) within the SR-region, and phosphorylated serine 364 and 367 (S364/367) within the C terminal region, respectively (Fig. 3h). Both domains have been shown to be relevant for the multimerization of N proteins, with the SR-region also being essential for replication^33^. Upon infection with HCoV-229E, a strong phosphorylation signal could be observed for both phosphorylation sites (Fig. 3i and Supplementary Fig. 3). Treatment with the JNK inhibitor SP600125, reduced N protein expression (Fig. 3j), while the phosphorylation ratio (p- S145/149/N Protein) of the remaining N Protein at the S145/149 sites was not significantly reduced following SP600125 treatment (Fig. 3k), suggesting this site can be phosphorylated by multiple kinases and does not specifically depend on JNK activity. In contrast, the phosphorylation ratio (p-S364/367/N Protein) at the S364/367 site was strongly reduced upon SP600125 treatment (Fig. 3l), providing evidence for a specific virus-induced activation of JNK that contributes to regulated N protein phosphorylation at these two sites (Fig. 3i and Supplementary Fig. 3). These data suggest that phosphorylation of the viral N protein by JNK supports the viral replication cycle.

### JNK inhibition lowers SARS-CoV-2 infectivity

**Fig. 4:**
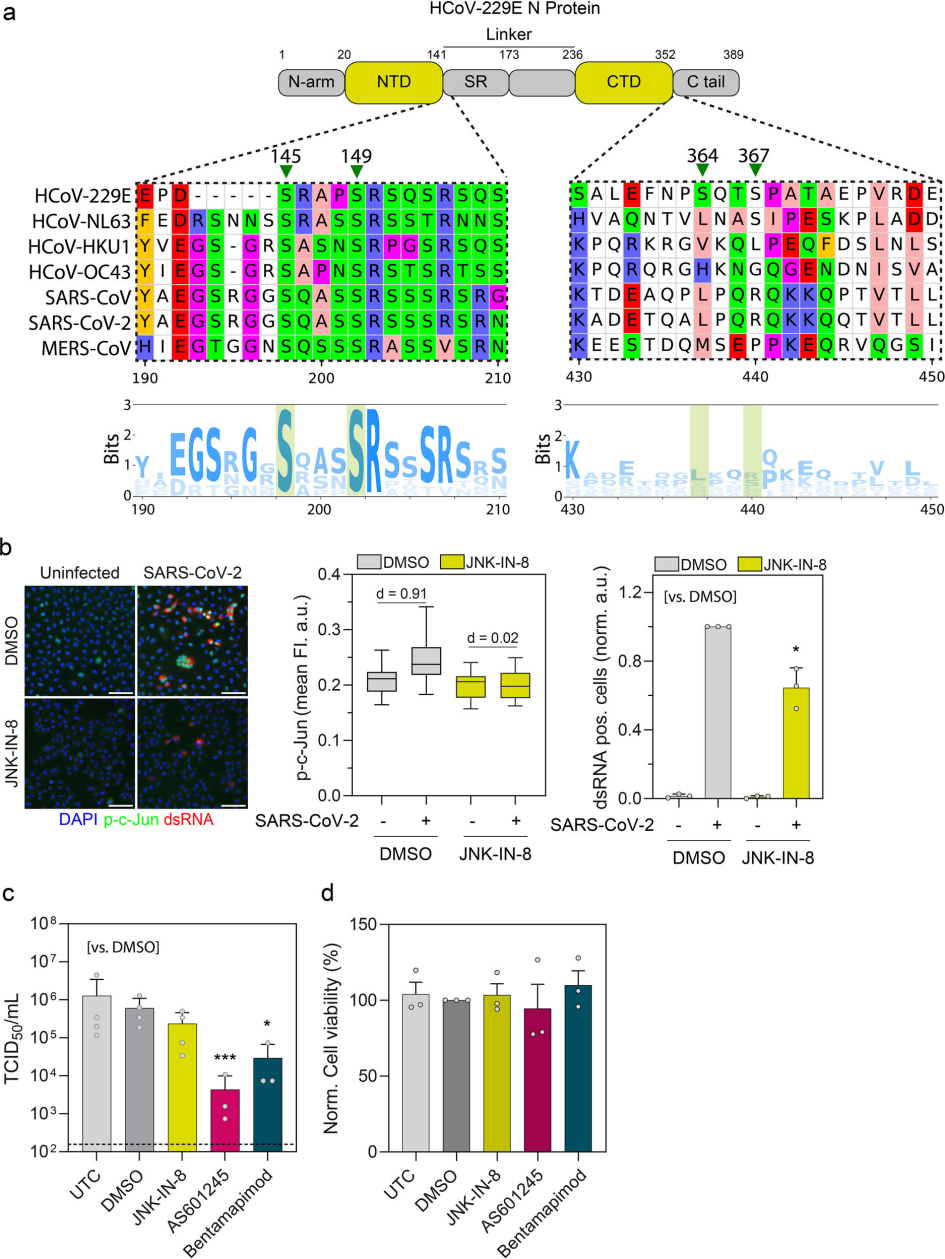
JNK inhibition lowers SARS-CoV-2 infectivity. **a** Top: Scheme of the domain structure of the HCoV-229E nucleocapsid protein (top) and multiple sequence alignment (middle), and sequence logo representation of multiple sequence alignment across six different human coronaviruses. **b** Left: Immunofluorescence images of double-stranded RNA (dsRNA) and phosphorylated c-Jun (p-c-Jun) in A549 A/T cells in uninfected control cells or 24 h after infection with SARS-CoV-2 (MOI 1) upon pretreatment with 10 µM JNK-IN-8 or DMSO. Nuclei were stained with DAPI. Scale bars = 100 µm. Middle: Boxplot of mean nuclear p-c-Jun fluorescence in single cells (1286 to 26,168 cells per condition from three independent experiments). The effect size was calculated as Cohen’s *d* (*d*). Right: Quantification of the normalized fraction of dsRNA-positive cells (mean ± SEM, *n* = 3). Statistical significance was determined using Welch’s *t*-test (*p < 0.05). **c** A549 A/T cells were either pretreated with different JNK inhibitors (all 10 µM), DMSO or left untreated (UTC) and subsequently inoculated with SARS-CoV-2 (MOI 1) for 1 h. Twenty-four hours post-infection, the supernatant was collected and viral titers determined by an endpoint dilution assay and calculated as TCID_50_/mL (mean ± SD, *n* = 3). Dashed lines indicate the lower limit of quantification (LLOQ). Statistical significance was determined using a one-way ANOVA with Dunnett’s post hoc test (***p < 0.001 and *p < 0.05). **d** Normalized cell viability in percent (%) upon for A549 A/T cells treated with different JNK inhibitors (all 10 µM), DMSO and untreated control cells.

To investigate the conservation of the two phosphorylation motifs within the HCoV-229E N protein, we compared the genomic sequence of the N proteins from all different human coronaviruses and determined the conservation of the respective serine residues (Fig. 4a). We noticed that the serine residues within the SR-rich domain (S145/149) were highly conserved among all viruses, while the serine residues close to the C terminus (S364/367) of the N protein were only present in the HCoV-229E N protein. Given the conservation of the serine residues within the SR-rich domain (S145/149) and previous evidence highlighting the significance of SR-rich domain phosphorylation for the coronavirus replication cycle^33–39^, we investigated whether JNK activity is also necessary during SARS-CoV-2 infection. Infection of human A549 lung epithelial cells expressing ACE2 and TMPRSS2 with SARS-CoV-2 revealed c-Jun phosphorylation in cells positive for dsRNA, implying JNK activation specifically within SARS-CoV-2-infected cells (Fig. 4b). Upon JNK-IN-8 treatment, c-Jun phosphorylation as well as the fraction of dsRNA-positive cells were strongly reduced, suggesting the activation and requirement of JNK kinase activity during SARS-CoV-2 infection. In agreement with this, all the JNKi JNK-IN-8, Bentamapimod and AS601245 lowered the production of infectious viruses (TCID50/mL—Fig. 4c), without compromising cell viability (Fig. 4d). Similar to our results for HCoV-229E, SARS-CoV-2 requires the JNK signaling for efficient replication.

## Discussion

The study of coronaviruses has become a crucial area of research, particularly given the significant global impact of outbreaks like SARS-CoV, MERS-CoV^1^, and most recently SARS-CoV-2^2^. Moreover, the common four human coronaviruses (229E, HKU1, NL63, and OC43) contribute to 15%–30% of cases of common colds in human adults and can cause severe disease in patients at risk (i.e. infants, elderly people, or immunocompromised patients)^3^. Consequently, understanding the host signaling pathways and characterizing virus-host interactions during the CoV infection cycle are essential for identifying functionally relevant host factors, which could potentially guide the development of novel and innovative therapies^5,32,40,41^. We observed strong activation of JNK specifically within HCoV-229E-infected cells. Pharmacological inhibition of JNK activity further reduced HCoV-229E infection and production of viral progeny production, suggesting an important role of JNK during HCoV-229E infection. Pattern recognition receptors (PRRs) such as RIG-I and MDA5 are essential cytosolic RNA sensors that detect viral infections by recognizing double-stranded RNA (dsRNA)^42,43^. Upon activation, these receptors trigger an innate immune response. In the context of viral infection, the activation of MAVS (mitochondrial antiviral-signaling protein) has been associated with the subsequent activation of the JNK pathway^44,45^. Hence, intracellular sensing of viral RNA during virus replication may promote JNK activation via MAVS independently of extracellular stimuli. Posttranslational modifications, particularly phosphorylation, are recognized as crucial modulators for the functionality and localization of viral proteins^29,31,46,47^. Although phosphorylation sites have been identified in various viral proteins, the N protein has been reported to exhibit the highest level of phosphorylation^31,32^. Using two phospho-specific antibodies, phosphorylation of the HCoV-229E N protein was detected in the serine-rich domain and the C-terminus. However, it remains unclear whether JNK itself acts as the kinase or if a kinase activated downstream of JNK is responsible for this process. Previous studies have shown that the phosphorylation status of the coronavirus N protein can modulate protein-protein and protein-RNA interactions^33–36^ as well as gel-liquid phase transition^39^ and viral transcription^37^. We observed no differences in the amount of viral RNA produced upon JNK inhibition (Fig. 3b), while the amounts of infectious progeny virus were greatly reduced (Fig. 2e), suggesting that JNK-dependent phosphorylation of the N protein is not required during viral replication, but rather be required during viral assembly and release. While the specific functions of individual phosphorylation sites are yet to be fully understood, the overall phosphorylation status of the nucleocapsid protein has been suggested to serve as a regulatory switch during the replication cycle^48^, between transcription and replication, or to promote genome packaging or unpackaging^38,39^. Future studies using reverse genetic systems to engineer viruses with desired mutations are required to study the role of individual phosphorylation sites during the CoV replication cycle^49,50^. Although our findings, in line with previous studies, suggest a potential role for JNK-mediated phosphorylation in the viral replication cycle, we cannot rule out the possibility that the inhibition of viral infection following JNK inhibition may be at least partially due to altered phosphorylation and/or expression of host or other viral proteins. In this context kinase substrate specificity mapping and in vitro phosphorylation assays could help to uncover protein kinases involved in the phosphorylation of the serine residues examined in this study (S145/149 and S364/367)^31^.

The strong conservation of the phosphorylation sites within the SR-region across human coronaviruses^31^ suggests a conserved mechanism that may be exploited for therapeutic purposes. The finding that inhibiting JNK activity reduces the infectivity of both HCoV-229E and SARS-CoV-2 indicates that the development of specific kinase inhibitors for clinical use may offer an avenue towards a broad-spectrum antiviral approach against multiple coronaviruses. The JNK signaling pathway comprises up to ten isoforms encoded by three genes, which are ubiquitously expressed and form a highly redundant core module responsible for maintaining cellular homeostasis and mounting rapid responses to various cellular stressors. Hence, global inhibition of JNKs using compounds that target the conserved ATP-binding pocket has shown limited clinical success. Future therapeutic strategies must adopt more targeted approaches. Potential promising directions could include (1) the development of allosteric inhibitors with isoform specificity, (2) small molecules designed to disrupt specific JNK-substrate interactions, such as those involving the well-characterized docking domains between JNK and its canonical substrate c-Jun^51,52^, and (3) tissue-specific downregulation of individual JNK isoforms using stabilized short RNA oligonucleotides^53^. In addition, proteolysis-targeting chimeras (PROTACs) offer an innovative strategy by enabling the transient degradation of selected JNK isoforms^54^. Another promising approach is the targeted delivery of JNK inhibitors to infected cells, for instance via topical administration to the nasopharyngeal epithelium during acute, symptomatic CoV infection. In summary, advancing JNK-targeted therapies will require a multimodal strategy that integrates the development of highly selective, potent compounds with a broad therapeutic index, refined dosing regimens, and precision delivery systems. Isoform-specific targeting and infection-contextual inhibition will be critical to maximize efficacy while minimizing off-target effects. Overall, our findings highlight the importance of studying the host-virus interplay to identify potential targets and mechanisms for developing host-directed antiviral therapies^5,32,40,41^. Further research is needed to clarify the specific functions of N protein phosphorylation in the replication cycles of different coronaviruses. This understanding could refine therapeutic strategies targeting kinase pathways, leading to more effective treatments for coronavirus infections, as well as other emerging or re-emerging viruses.

## Methods

### Plasmids

pLentiPGK Puro DEST JNK KTR AA Clover (Addgene plasmid #90238) and pLentiPGK Puro DEST JNK KTR Clover (Addgene plasmid # 59151) were a gift from Markus Covert. pCMVR8.74 (Addgene plasmid # 22036) and pMD2.G (Addgene plasmid # 12259) were a gift from Didier Trono. pLVX-M-puro (Addgene plasmid #125839) was a gift from Boyi Gan. 3xAP1pGL3 (Addgene plasmid #40342) and pMIEG3-JunDN (Addgene plasmid #40350) were a gift from Alexander Dent. pCMVR8.74 (Addgene plasmid # 22036) and pMD2.G (Addgene plasmid # 12259) were a gift from Didier Trono. pLVX-AP1-GFP and pLVX-JunDN-HA were cloned using Gibson assembly (New England Biolabs (NEB), E2611L) with inserts amplified with appropriate overhangs using Q5 high-fidelity DNA polymerase (NEB, M0491L).

### Reagents

JNK and c-Jun inhibitors were obtained from MedChemExpress (MCE): JNK-IN-8 (HY-13319), Bentamapimod (HY-14761), SP600125 (HY-12041), AS601245 (HY-11010), T-5224 (HY-12270), SR 11302 (HY-15870), 1-Methyl-6-oxo-1,6-dihydropyridine-3-carboxylic acid (HY-N4226). Recombinant Human TNF-α (300-01A) was obtained from Preprotech. Human recombinant IL-1a was a kind gift from Jeremy Saklatvala (Oxford, UK) or was prepared in our laboratory as described and used at 10 ng/mL final concentration in all experiments^55^. Anti-NSP8 was a gift from John Ziebuhr, Gießen, Germany. The antibodies against SS145/147 and SS364/367 were generated using a commercial immunization program by the company Eurogentec (4102 Seraing, Belgium). Briefly, rabbits were immunized with phosphorylated synthetic peptides h- C + EEPD-S_(PO3H2)_-RAP-S_(PO3H2)_-RSQ -nh2 (for SS145/149) or h- C + EFNP-S*(PO3H2)*-QT-S*(PO3H2)*-PATA -nh2 (for SS364/367). The sera of two rabbits were analyzed for high-titer antibodies against the phosphorylated forms of the peptides compared to the carrier by specific ELISA with P-peptide-coated plates. The final bleed of one rabbit with the highest titer was used to purify high-titer P-specific antibodies from serum. First, specific antibodies were purified by P-peptide affinity chromatography. Second, any remaining IgG fraction also recognizing the unmodified peptide structures was removed by a second purification step on affinity matrices coupled to the unmodified peptides. The flowthrough of this column contained highly specific P-peptide antibodies with titers >1 × 10^4^ for the P-peptide.

### Cell culture

Huh7 cells were cultured in Dulbecco’s Modified Eagle’s Medium (DMEM) supplemented with 10% (v/v) fetal calf serum (FCS), 1% (v/v) non-essential amino acids (NEAA), 100 IU/mL penicillin, 100 μg/mL streptomycin, and 2 mM L-glutamine. ACE2 and TMPRSS2 overexpressing A549 (A549-A/T) cells were cultured in DMEM with 5% (v/v) FCS, 1% (v/v) NEAA, 100 IU/mL penicillin, 100 μg/mL streptomycin, and 2 mM L-glutamine. The cells were additionally selected with Blasticidin (10 µg/mL) and Puromycin (0.5 µg/mL).

### Production of ectopically expressing cell lines via lentiviral transduction

For the production of lentiviral particles, 4 × 10^5^ 293 T cells were seeded on collagen-coated 6-well plates. The following day, the 293 T cells were transfected with the plasmids pcz-VSV-G, pCMV-dR8.74, along with plasmids encoding the desired transgenes using Lipofectamine 2000 (Invitrogen, Cat. 40 Nr. 11668019) following the manufacturer’s instructions. Six hours post-transfection, the medium was changed, and lentiviral particles were harvested 48 h post-transfection. Supernatants were filtered (Filtropur 0.45, Sarstedt, Cat. Nr. 83.1826) and supplemented with HEPES and polybrene, and either used directly or stored at −80 °C. For transduction, Huh7 cells were seeded on a 6-well plate and inoculated with 1 mL of lentiviral particles for 6–8 h. Selection of the transduced cells was started 48 h post-transduction using 2.5 μg/mL puromycin. Transgene expression was validated via fluorescence microscopy.

### Virus infection assays

Huh7 or A549-A/T cells were seeded at a density of 8 × 10^4^ cells/well in a 24-well plate. After cell attachment, cells were pretreated with different inhibitors for at least 30 min. For infection, Huh7 cells were inoculated with human coronavirus 229E (MOI 0.1)^56^ in 10% FCS-containing DMEM for 1 h, while A549-A/T were inoculated with hCoV-19/Germany/BY-Bochum-1/2020 (B.1.1.70; GISAID accession ID: EPI_ISL_1118929, MOI 1) for 1 h (A549-A/T cells). Hereafter, the cells were washed three times with 1 × PBS and treated again with the respective inhibitors and immunosuppressants as indicated in the figure legends.

### AP1 reporter assay

Huh7 cells stably expressing the AP1-GFP reporter were seeded into 96-well plates (10⁴ cells per well). Twenty-four hours later, the culture medium was removed, and cells were incubated with 50 µL of either DMSO or c-Jun inhibitors at a concentration of 50 µM for 1 h. Following this pre-treatment, 50 µL of medium containing IL-1α was added to each well, resulting in a final concentration of 10 ng/mL IL-1α and 25 µM inhibitor. After 24 h of stimulation, cells were fixed with paraformaldehyde (PFA), and nuclei were counterstained with DAPI prior to imaging. GFP intensity in the nucleus was determined using CellProfiler.

### siRNA transfection

Huh7 cells (5 × 10^4^ cells/well) were seeded in 24-well plates and transiently transfected with 10 nM of siRNAs targeting JNK1 (Thermo Fischer Scientific, s11153) and JNK2 (Thermo Fischer Scientific, s1452), and one control non-targeting siRNA (Thermo Fischer Scientific, 4390846) using Lipofectamine RNAiMAX (Invitrogen, #13778) following the manufacturer´s instructions. Two days post-transfection (d.p.t.), cells were either infected with HCoV-229E (MOI 0.1—see Virus infection assays) or 3 d.p.t. lysed for western blot analysis.

### Immunoblotting

Whole cell extracts were prepared in Triton cell lysis buffer (10 mM Tris, pH 7.05, 30 mM NaPPi, 50 mM NaCl, 1% Triton X-100, 2 mM Na3 VO 4, 50 mM NaF, 20 mM ß-glycerophosphate and freshly added 0.5 mM PMSF, 2.5 μg/mL leupeptin, 1.0 μg/mL pepstatin, 1 μM microcystin). Cell lysates were subjected to SDS-PAGE on 7-12.5% gels. Proteins were separated on SDS-PAGE and electrophoretically transferred to PVDF membranes (Roth, Roti-PVDF (0.45 μm)). After blocking with 5% dried milk in Tris-HCl-buffered saline/0.05% Tween (TBST) for 1 h, membranes were incubated for 12–24 h with primary antibodies, including custom made anti-phospho N Protein HCoV-229E Serin 145/149 (Eurogentec), anti-phospho N Protein HCoV-229E Serin 364/367 (Eurogentec), anti-phospho JNK Threonine 183/Tyrosine 185 (Cell Signaling (#9251)), anti-JNK (Santa Cruz (#sc-571)), anti-Jun (Santa Cruz (#sc-1694)), anti-N Protein HCoV-229E (Ingenasa (Batch 250609)), anti-β-Actin (Santa Cruz (#sc-4778)). Afterwards, membranes were washed in TBST and incubated for 1–2 h with the peroxidase-coupled secondary antibody. Proteins were detected by using enhanced chemiluminescence (ECL) systems from Millipore or GE Healthcare. Images were acquired and quantified using a Kodak Image Station 440 CF and the software Kodak 1D, 3.6, or the ChemiDoc Touch Imaging System (BioRad) and the software ImageLab, V_5.2.1 or higher (Bio-Rad).

### Immunofluorescence

Cells were fixed in 3% paraformaldehyde at room temperature for at least 10 min and washed three times with PBS. Permeabilization was achieved using 0.2% Triton X-100 for 5 min, followed by three additional PBS washes. Cells were blocked in 5% horse serum in PBS for 1 h at room temperature. Primary antibodies were diluted in 5% horse serum-PBS and incubated overnight at 4 °C. Anti-phospho-c-Jun (Ser73) antibody (1:500, Cell Signaling Technology #9164), anti-N protein (1:1000, Anticuerpo Monoclonal, 1E7, Ingenasa), HA Tag Monoclonal Antibody (1:500, Thermo Fisher 2-2.2.14) and anti-dsRNA antibody (1:1000, SCICONS 10010500) were used as primary antibodies. Secondary antibodies were Alexa 488-labeled donkey anti-rabbit IgG (Invitrogen A11008), Alexa 555-labeled donkey anti-mouse IgG (Invitrogen A11008) or Alexa 488-labeled donkey anti-mouse IgG (Invitrogen A21206), used at 1/1000 dilution in 5% horse serum-PBS, and incubated for 1–2 h at room temperature in the dark. Nuclei were stained with DAPI (1 μg/mL).

### Microscopy and image analysis

Immunofluorescence images were acquired with a wide-field fluorescence microscope (Keyence BZ-X800E) using 4x, 10x and 20x objectives and BZ-X Filter DAPI, BZ-X Filter GFP and BZ-X Filter TRITC. To determine the fractions of dsRNA-positive cells, DAPI-stained nuclei were segmented and expanded by 3 pixels. Spots of dsRNA were detected within the expanded nuclei to identify infected cells using CellProfiler^57^. For JNK KTR cells, segmentation and object quantification were performed with CellProfiler^57^. Nucleus and a 5-pixel wide cytoplasm ring (cytoring) were segmented using DAPI-stained nuclei, and intensities were quantified in the KTR channel. Mean fluorescence intensities of nuclear p-c-Jun were obtained after segmentation of DAPI-stained nuclei using CellProfiler^57^. Live cell imaging experiments, confocal images were acquired on a CQ1 Confocal Imaging Cytometer (Yokogawa) using a 20x objective in combination with a BP447/60 (Hoechst) and BP525/50 (Clover) filters. A total of 1 × 10^4^ cells/well Huh7-JNK-KTR cells were seeded in 96-well plates. Nuclei of living cells were stained with Hoechst were acquired in a 30 min interval over 24 h following infection with HCoV-229E and non-treated control cells.

### Cell viability assays

Cell viability was determined by adding 0.5 mg/mL 3-(4,5-dimethylthiazol-2-yl)-2,5-diphenyltetrazolium bromide (MTT) (Sigma) substrate to cells and subsequent incubation at 37 °C and 5% CO_2_ for 1–2 h. Medium was removed and 50 μL of DMSO was added to each well. The absorbance of each well was read on a microplate absorbance reader (Tecan Group Ltd, Männedorf, Switzerland) at 570 nm. Cells treated with 70% ethanol for 10 min served as background control.

### Multiple sequence alignment

Reference proteomes of viruses from the *Coronaviridae* family were downloaded from UniProt. N protein sequences (HCoV-229E - P15130; HCoV-NL63 - Q6Q1R8; SARS-CoV - P59595; MERS-CoV - T2BBK0; HCoV-OC43 - P33469; HCoV-HKU1 - Q19U25; SARS-CoV-2 - P0DTC9) were aligned to the HCoV-299E N protein with CLUSTAL 2.1 Multiple Sequence Alignments (MSA). Data visualization was conducted using a Jupyter Notebook 7.0.3 (Python 3.10.10) in combination with MsaViz (pyMSAviz package) and SeqIO (BioPython library) to visualize MSA and create consensus plots. The logomaker library was used to create conservation plots.

### Software

Data visualization and statistical analysis were performed using GraphPad Prism v10. Dose-response curves were calculated using a four-parameter non-linear regression model implemented in GraphPad Prism v10. Fluorescence microscopy images were analyzed with Fiji and/or CellProfiler^57,58^. The effect size was calculated as Cohen’s *d* (*d*). An Alpha-fold model of the HCoV-229E nucleocapsid (GenBank: QNT54801.1) was processed and displayed with ChimeraX (Version: 1.6rc202304072249 (2023-04-07)).

## Supplementary information

Supplementary Information

Supplementary Movie

## Data Availability

The datasets used and/or analyzed during the current study are available from the corresponding authors on reasonable request.

## Code availability

The underlying code for this study is not publicly available but may be made available to qualified researchers on reasonable request from the corresponding author.
