# Supplementary material for: JNK kinase regulates phosphorylation of HCoV-229E nucleocapsid protein
Source: Npj Viruses. 2025 Sep 18;3:69. doi: 10.1038/s44298-025-00152-7 (PMC12446446; doi:10.1038/s44298-025-00152-7)
Supplement: Supplementary file 1 — Supplementary Information [file 44298_2025_152_MOESM1_ESM.pdf]

Supplementary information

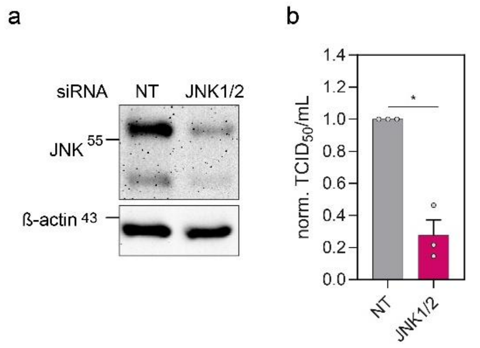

Supplementary figure 1: (a) Lysates of Huh7 cells 48 h after transfection with either non-targeting siRNA (NT) or siRNA targeting JNK1/2 were immunoblotted for JNK and ß-actin. (b) Huh7 cells were either transfected with non-targeting (NT) siRNA or siRNA targeting JNK1/2 and 48 h after transfection inoculated with HCoV-229E (MOI 0.1) for 1 h. Twenty-four hours post infection the supernatant was collected and viral titers determined by an end point dilution assay and calculated as normalized (norm.) $TCID_{50}$/mL (mean ± SD, n = 3). Statistical significance was determined using Welch's t-test (*P <0.05).

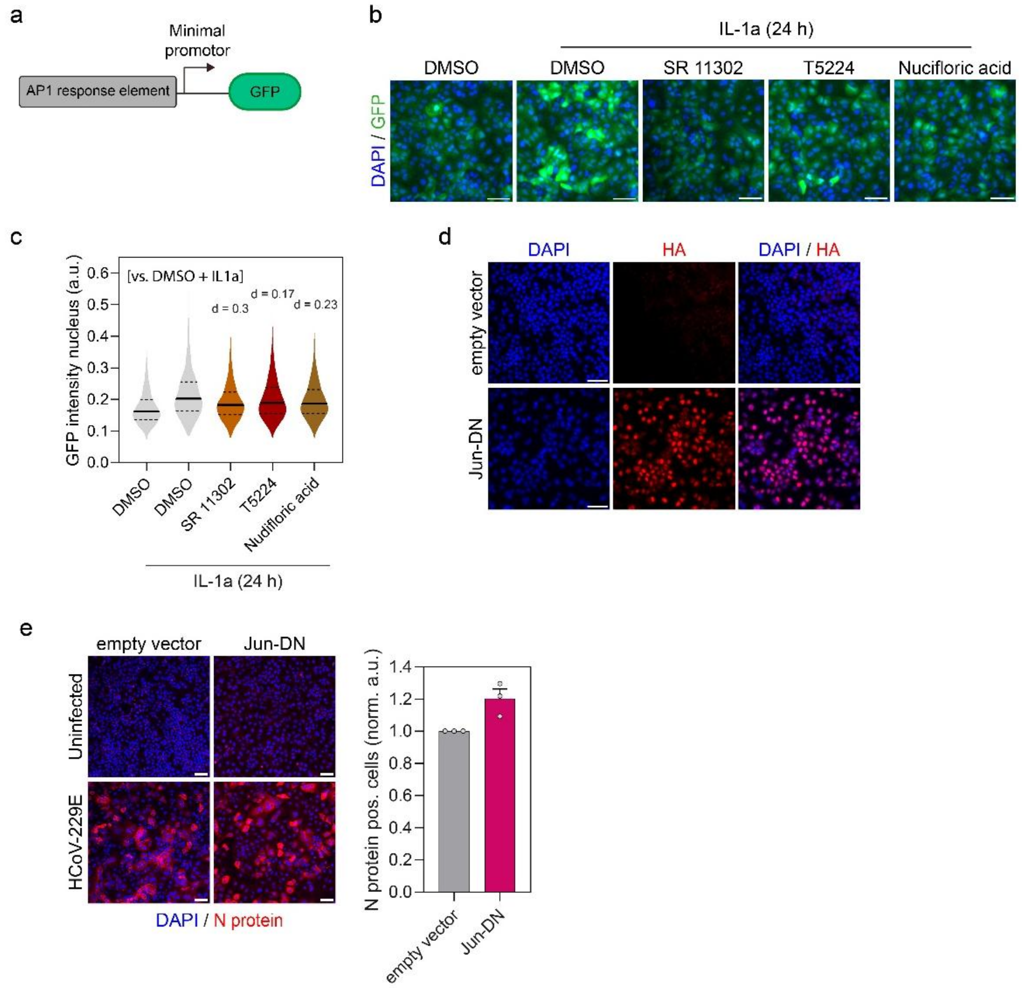

Supplementary figure 2: (a) Schematic of the AP1-GFP reporter construct. (b) Huh7 cells stably transduced with the AP1-GFP reporter were preincubated with different c-Jun inhibitors (all 25 µM) or DMSO and subsequently stimulated with 10 ng/mL IL-1α for 24 h. (c) Quantification of AP1-GFP activation upon IL-1α treatment and in untreated control cells (3179 to 3941 cells per condition). The effect sizes were calculated as Cohen´s d (d). (d) Huh7 cells stably transduced with empty vector control or a dominant negative Jun variant (Jun-DN) were stained with anti-HA antibody. Nuclei were stained with DAPI. (e) Left: Huh7 cells stably transduced with empty vector control or Jun-DN 24 h after infection with infection with HCoV-229E (MOI 0.1) and uninfected control cells were stained for the HCoV-229E nucleocapsid (N protein). Nuclei were stained with DAPI. Right: Quantification of the normalized fraction of N protein positive cells (mean ± SD; n = 3). All Scale bars = 100 µm.

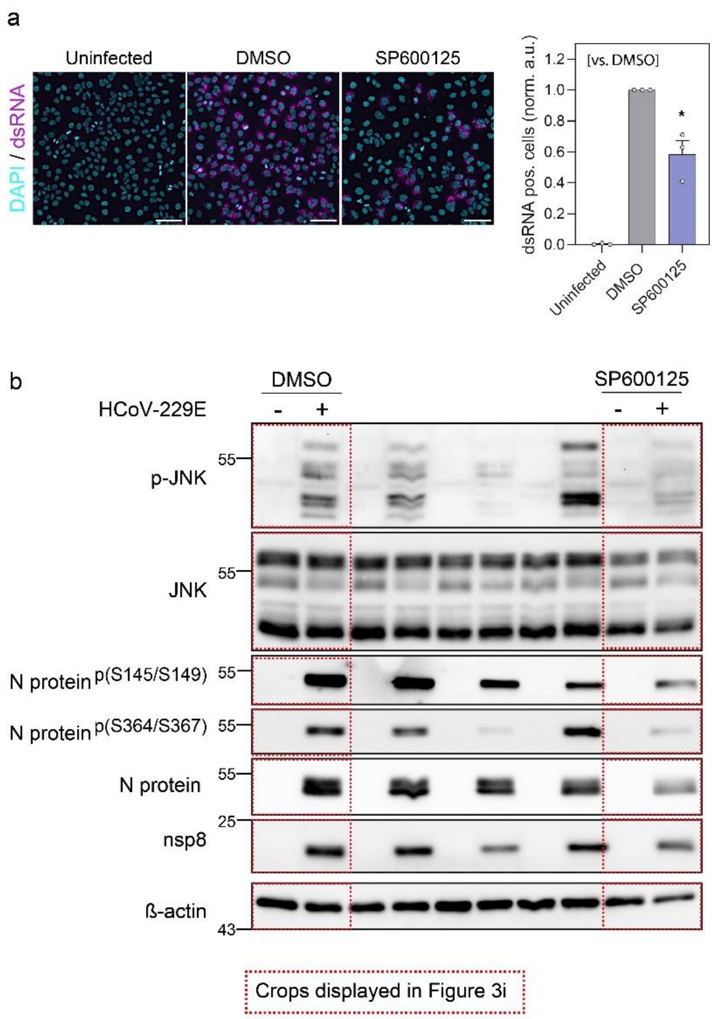

Supplementary figure 3: (a) Left: Immunofluorescence images of double stranded RNA (dsRNA) in uninfected Huh7 cells or 24 h after infection with HCoV-229E (MOI 0.1) upon pretreatment with 20 µM SP600125 or DMSO. Nuclei were stained with DAPI. Scale bars = 100 µm. Right: Quantification of the normalized fraction of dsRNA-positive cells (mean ± SEM; n = 3). Statistical significance was determined using Welch's t-test (*P <0.05). (b) Uncropped western blots. Red crops are shown in Figure 3i.

Supplementary video 1: Huh7 cells expressing JNK-KTR-Clover. Uninfected control cells (left) and cells following infection with HCoV-229E (MOI 1 right).
